# Unilateral Acute Macular Toxoplasmic Chorioretinitis Associated with White Dot-Like Choroidal Involvement Demonstrated on Indocyanine Green Angiography

**DOI:** 10.4274/tjo.galenos.2020.93636

**Published:** 2020-08-26

**Authors:** Şefik Can İpek, Pınar Çakar Özdal, Salih Kavukçu, Ali Osman Saatci

**Affiliations:** 1 Dokuz Eylül University Faculty of Medicine, Department of Ophthalmology, İzmir, Turkey; 2 University of Health Sciences Turkey, Ulucanlar Eye Training and Research Hospital, Ankara, Turkey; 3 Dokuz Eylül University Faculty of Medicine, Department of Pediatrics, Division of Pediatric Nephrology, İzmir, Turkey

**Keywords:** White dot syndrome, indocyanine green angiography, chorioretinitis, optical coherence tomography angiography, toxoplasmosis

## Abstract

A 9-year-old otherwise healthy boy was examined due to a 4-day history of visual decline in his right eye. Ophthalmological examination revealed an area of active retinochoroiditis in the right macula. Indocyanine green angiography (ICGA) demonstrated multiple hypocyanescent dots surrounding the active lesion extending 360 degrees towards the equator. Optical coherence tomography angiography (OCTA) exhibited dark dots on the choriocapillaris slab over areas corresponding to the hypocyanescent dots detected with ICGA. Full systemic examination and laboratory investigations were carried out. *Toxoplasma gondii* serology was positive. The diagnosis of toxoplasmic chorioretinitis with white dot-like choroidal involvement was made. Trimethoprim/sulfamethoxazole, azithromycin, and oral prednisolone were administered orally. On repeated ICGA 2 weeks later, the scattered hypocyanescent dots were significantly fewer in number. A month later, right visual acuity was improved, the macular chorioretinitis focus had become inactive, an epiretinal membrane had formed, and the dark dots on the choriocapillaris slab of OCTA were markedly diminished. ICGA may be helpful to observe possible, subtle choroidal involvement in patients with toxoplasmic chorioretinitis.

## Introduction

Toxoplasmic chorioretinitis is the most common type of infectious uveitis. Several atypical findings may accompany chorioretinitis such as scleritis, Fuchs’-like anterior uveitis, punctate outer retinitis, necrotizing retinitis, Coats’-type response, branch retinal artery occlusion, frosted branch angiitis-like retinal vasculitis, choroidal neovascular membrane, retinal detachment, papillitis, neuroretinitis, and retrobulbar neuritis. We herein report a 9-year-old boy with unilateral macular toxoplasmic chorioretinitis in association with multiple satellite dot-like choroidal involvement.

## Case Report

**Figure 1:**
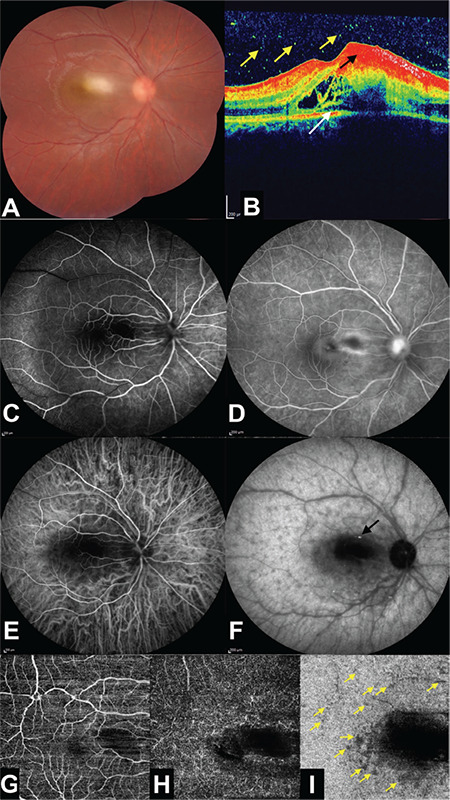
Right eye at presentation. A) Composite color photograph: hazy view of the posterior pole and central lesion of active chorioretinitis. B) Optical coherence tomographic section: hyperreflective vitreous cells (yellow arrows), increased reflectivity of the inner retina corresponding to the lesion (black arrow), and intraretinal fluid and subtle subretinal fluid (white arrow). C) Fluorescein angiogram, arteriovenous phase: hypofluorescence of the lesion. D) Fluorescein angiogram, late venous phase: staining of the lesion borders and nearby vessels. E) Indocyanine green angiogram, early phase: faint hypocyanescent macular lesion. F) Indocyanine green angiogram, late phase: prominent hypocyanescent central lesion, multiple hypocyanescent dots extending 360-degrees towards the equator, and a focus of hypercyanescence of the adjacent vessel (most likely a Kyrieleis plaque) (black arrow). G) Optical coherence tomography angiography, superficial capillary plexus slab. H) Optical coherence tomography angiography, deep capillary plexus slab. I) Optical coherence tomography angiography, choriocapillaris slab: central dark area and perimacular dark small dots (yellow arrows)

**Figure 2:**
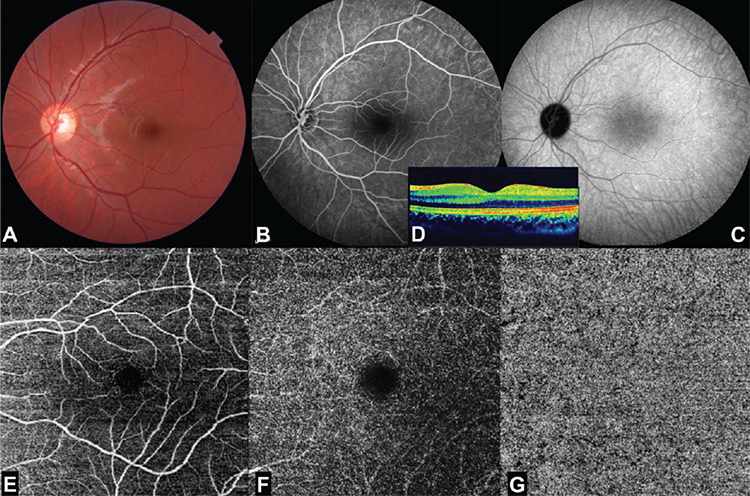
Left eye at presentation. A) Color photograph: normal fundus appearance. B) Fluorescein angiogram, venous phase: normal angiogram. C) Indocyanine green angiogram, late phase: normal angiogram. D) Optical coherence tomographic section: normal macular contour. E, F, G) Optical coherence tomography angiography; superficial capillary plexus, deep capillary plexus and choriocapillaris slab; respectively. Normal appearance

A 9-year-old otherwise healthy boy with a 4-day history of visual decline in his right eye was examined. Best-corrected visual acuity (BCVA) was 20/200 in the right eye and 20/20 in the left eye. While slit-lamp examination was normal in the left eye, there were +2 cells in the right anterior chamber with mild to moderate vitritis. Fundus examination of the right eye revealed a macular yellow-whitish area of infiltration with ill-defined borders (Figure 1A). Optical coherence tomography (OCT) (Spectralis; Heidelberg Engineering, Heidelberg, Germany) of the right eye delineated hyperreflective dots in the vitreous cavity, intraretinal fluid, subtle subretinal fluid, and hyperreflective inner retinal area with backshadowing corresponding to the chorioretinitis site (Figure 1B). Fluorescein angiography (FA) (Spectralis; Heidelberg Engineering, Heidelberg, Germany) of the right fundus demonstrated that the main central lesion was hypofluorescent throughout the angiogram with gradual staining of its borders and the adjacent vessels (Figure 1C, D). Indocyanine green angiography (ICGA) (Spectralis; Heidelberg Engineering, Heidelberg, Germany) showed that the main lesion was hypocyanescent most notably in the late phases of the angiogram. ICGA revealed a multitude of hypocyanescent dots surrounding the active lesion extending 360 degrees towards the equator and a focus of hypercyanescence in the nearby vessel most likely corresponding to a Kyrieleis plaque (Figure 1E and F). OCT angiography (OCTA) (Topcon DRI OCT Triton, Topcon, Japan) (6x6 mm) exhibited a central dark area and perimacular small dark dots noticed especially on the choriocapillaris slab in sites corresponding to the satellite hypocyanescent dots noticed on ICGA (Figure 1G, H, I). The left fundus was normal (Figure 2A). Fluorescein, ICGA, OCT, and OCTA examinations of the left eye were also normal (Figure 2B, C, D, E, F, G). Full systemic examination and laboratory investigations were carried out. Serology for *Toxoplasma gondii* in serum showed an immunoglobulin (Ig)G level of 38.6 IU/mL and IgM antibodies were negative. Oral trimethoprim/sulfamethoxazole (4 mg/20 mg/kg/day for 35 days), azithromycin (10 mg/kg/day for 15 days), and prednisolone (20 mg/day with a gradual taper) together with topical prednisolone acetate 1% (6 times a day with a gradual taper) were administered.

**Figure 3:**
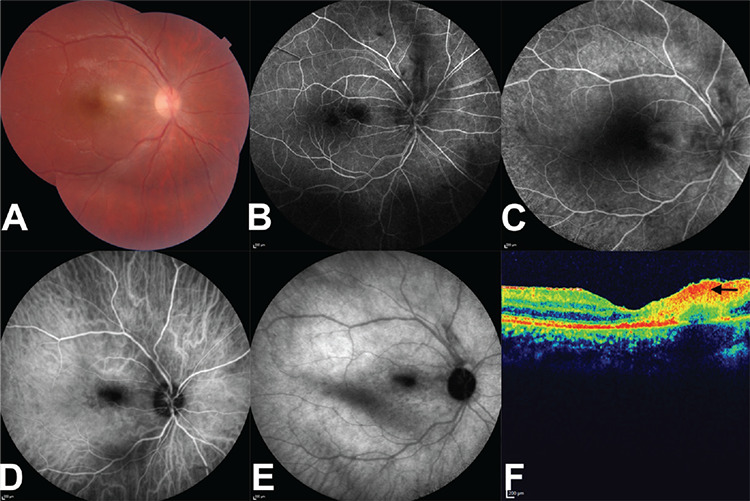
Right eye at 2 weeks. A) Composite color photograph: macular lesion size was diminished. B) Fluorescein angiogram, arteriovenous phase: shrunken central hypofluorescent lesion and the masking effect of some vitreous opacities. C) Fluorescein angiogram, late venous phase: faint border staining of the hypofluorescent lesion site. D) Indocyanine green angiogram; early phase, faint hypocyanescence of the macular lesion. E) Indocyanine green angiogram, late phase: decreased number of scattered hypocyanescent dots, Kyrieleis plaque was no longer visible. F) Optical coherence tomographic section: mild intraretinal fluid, shrunken hyperreflective inner retinal area with less backshadowing (black arrow)

Two weeks later, the lesion became smaller (Figure 3A) and FA and ICGA were repeated (Figure 3B, C, D, E). The hypofluorescent/hypocyanescent area on FA/ICGA was markedly smaller and the hypocyanescent dots seen on the first ICGA were fewer in number. OCT showed that the intraretinal fluid was dramatically less and the hyperreflective inner retinal area was smaller in size with a slightly smaller area of backshadowing (Figure 3F).

**Figure 4:**
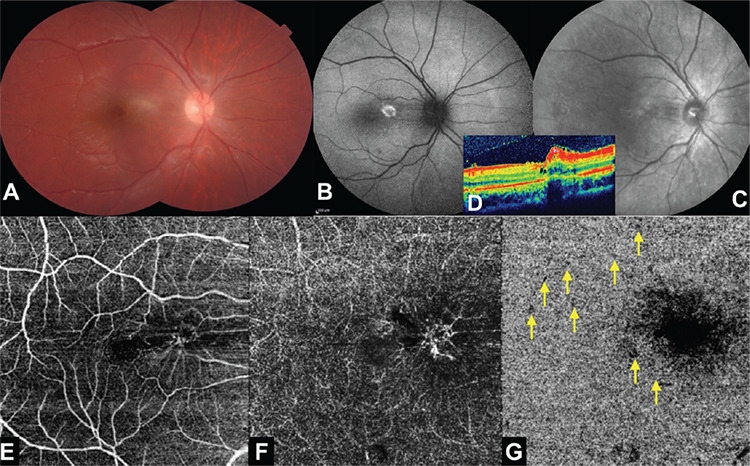
Right eye at 1 month. A) Composite color photograph; smaller and inactive-appearing central lesion and a tiny epiretinal membrane formation. B) Fundus autofluorescent image: hyperautofluorescence of the original lesion site. C) Reflectance image: faint appearance of the lesion site. D) Optical coherence tomographic section: markedly improved foveal architecture with a tiny epiretinal membrane on top of the residual hyperreflective area. E) Optical coherence tomography angiography, superficial capillary plexus slab. F) Optical coherence tomography angiography, deep capillary plexus slab. G) Optical coherence tomography angiography, choriocapillaris slab: less prominent central dark area and a few persistent dark dots surrounding the macula (yellow arrows)

A month later, the patient’s BCVA in the right eye was 20/50. Fundus examination showed that the inflammatory chorioretinal lesion site appeared nearly inactive and smaller in size (Figure 4A). While fundus autofluorescence images showed hyperautofluorescence of the residue of original inflammation site (Figure 4B), the lesion was barely discernible on a reflectance image (Figure 4C). OCT showed that the retinal layers were reorganized dramatically but a tiny epiretinal membrane formation was present (Figure 4D). OCTA exhibited some persistent dark dots on the choriocapillaris slab with the reflection of the pucker (Figure 4E, F, G). A third FA and ICGA were not obtained as the patient improved both clinically and anatomically.

## Discussion

Satellite dark dots in the choroid can be noted in eyes with active ocular toxoplasmosis.^1,2,3^ Knecht et al.^4^ argued that there was a secondary choriocapillaritis due to an inflammatory reaction of the choriocapillaris in the vicinity of the infection or inflammatory foci of the retina or choroid in many infectious uveitis entities including toxoplasmic chorioretinitis. They also speculated that satellite dark dots detected on ICGA might represent a temporary occlusion of the choriocapillaris likely related to a nonmechanical, cytokine-induced capillary occlusion.

Atmaca et al.^5^ retrospectively reviewed the charts and angiograms of 21 patients with ocular toxoplasmosis and noted hypofluorescent satellite dark dots adjacent to the main lesion in 11 of the eyes with active retinochoroiditis and they believed that hypersensitivity reaction might have played a role in the occurrence of these dots. However, the dark dots noted by Atmaca et al.^5^ did not present in a widespread white dot-like choroidal distribution as in our case. To our knowledge, 360-degree widespread white dot-like choroidal involvement observed in the present case has not been documented before, and we also shared the OCTA images of these dots.

In the present case, the 360-degree scattered satellite choroidal dots caught our attention only after viewing the sequence of ICGA, as those dots were very subtle both on color fundus photography and FA. Therefore, we believe that ICGA may be helpful to detect the presence of such satellite choroidal changes in patients with toxoplasmic chorioretinitis. These circumferentially scattered dark dots could be seen especially on the choriocapillaris slab of OCTA and they might either reflect impaired choroidal circulation or represent blockage due to inflammation-related coexistent subclinical inflammation or signs of a hypersensitivity reaction.
